# Comparison of primary care models in the prevention of cardiovascular disease - a cross sectional study

**DOI:** 10.1186/1471-2296-12-114

**Published:** 2011-10-18

**Authors:** Clare Liddy, Jatinderpreet Singh, William Hogg, Simone Dahrouge, Monica Taljaard

**Affiliations:** 1 C.T. Lamont Primary Health Care Research Centre, Élisabeth Bruyère Research Institute, 43 Bruyère St. Ottawa, Ontario, K1N 5C8, Canada; 2 Department of Family Medicine, University of Ottawa, Room, 43 Bruyère St. Ottawa, Ontario, K1N 5C8, Canada; 3 Clinical Epidemiology Program, Ottawa Hospital Research Institute, 1053 Carling Ave., Ottawa, Ontario, K1Y 4E9, Canada; 4 Department of Epidemiology and Community Medicine, University of Ottawa, Room 3105, 451 Smyth Road, Ottawa, Ontario, K1H 8M5, Canada

## Abstract

**Background:**

Primary care providers play an important role in preventing and managing cardiovascular disease. This study compared the quality of preventive cardiovascular care delivery amongst different primary care models.

**Methods:**

This is a secondary analysis of a larger randomized control trial, known as the Improved Delivery of Cardiovascular Care (IDOCC) through Outreach Facilitation. Using baseline data collected through IDOCC, we conducted a cross-sectional study of 82 primary care practices from three delivery models in Eastern Ontario, Canada: 43 fee-for-service, 27 blended-capitation and 12 community health centres with salary-based physicians. Medical chart audits from 4,808 patients with or at high risk of developing cardiovascular disease were used to examine each practice's adherence to ten evidence-based processes of care for diabetes, chronic kidney disease, dyslipidemia, hypertension, weight management, and smoking cessation care. Generalized estimating equation models adjusting for age, sex, rurality, number of cardiovascular-related comorbidities, and year of data collection were used to compare guideline adherence amongst the three models.

**Results:**

The percentage of patients with diabetes that received two hemoglobin A1c tests during the study year was significantly higher in community health centres (69%) than in fee-for-service (45%) practices (Adjusted Odds Ratio (AOR) = 2.4 [95% CI 1.4-4.2], p = 0.001). Blended capitation practices had a significantly higher percentage of patients who had their waistlines monitored than in fee-for-service practices (19% vs. 5%, AOR = 3.7 [1.8-7.8], p = 0.0006), and who were recommended a smoking cessation drug when compared to community health centres (33% vs. 16%, AOR = 2.4 [1.3-4.6], p = 0.007). Overall, quality of diabetes care was higher in community health centres, while smoking cessation care and weight management was higher in the blended-capitation models. Fee-for-service practices had the greatest gaps in care, most noticeably in diabetes care and weight management.

**Conclusions:**

This study adds to the evidence suggesting that primary care delivery model impacts quality of care. These findings support current Ontario reforms to move away from the traditional fee-for-service practice.

**Trial Registration:**

ClinicalTrials.gov: NCT00574808

## Background

Many industrialized nations have initiated reforms to optimize their delivery of primary care, as increasing evidence continues to demonstrate the important role that primary care plays in preventing and managing chronic health conditions [1-3].

Over the past decade, several Canadian provinces have implemented new models of primary health care delivery, with many of these approaches focussing on alternative physician payment models and placing greater emphasis on the development of multidisciplinary health care teams [4]. The resulting primary care landscape in provinces such as Ontario is highly diverse, as practices operate under different funding models and team structures which now coexist within the province.

Much of the focus in Ontario has been on establishing practices in which physicians are paid principally by capitation, a payment system based on the number and type of patients enrolled [5]. These practices also derive a smaller component of their earnings from fee-for-service activities and incentive payments for reaching certain clinical targets or for delivering specified health promotion activities [5,6]. In the latter part of the decade, the province invested in the development of interprofessional health care teams within these capitation payment models. The Ontario Government provides funding for practices operating under these models to support allied health professionals such as nurse practitioners, pharmacists, social workers, and dieticians [5].

About a quarter of the family physicians in Ontario practice in primary care delivery models which employ a blended capitation remuneration approach [5]. Since remuneration is dissociated from the number of patient encounters, this approach to primary care is expected to encourage health promotion activities as well as targeted care programs for high risk patients. Although many governments continue to promote organizational models that employ capitation and multidisciplinary care, few studies have compared the quality of chronic disease care being delivered in different primary care models [7].

As part of a larger quality improvement project focused on cardiovascular care delivery in primary care, we were able to evaluate and contrast the preventive cardiovascular disease care being delivered in family practices that operate under different primary care organizational models to determine whether the practices differ in their quality of care. This article describes the results of this secondary analysis of baseline medical chart data from 82 primary care practices in Ontario, Canada.

## Methods

### Study Design

We conducted a cross-sectional study comparing the quality of preventive cardiovascular care in three primary care models currently available in Ontario, Canada: fee for service (FFS), blended capitation, and community health centres in which the physicians are salary-based. We used baseline data collected through the Improved Delivery of Cardiovascular Care (IDOCC) through Outreach Facilitation project http://www.idocc.ca, a quality improvement project [8].

IDOCC is a stepped wedge cluster randomized control trial where Outreach Facilitators work with primary care practices to optimize cardiovascular disease prevention and management in patients at highest risk. A stepped wedge design is a type of crossover study in which different clusters cross over from the control arm to the intervention arm at different time points [9]. The IDOCC program is being offered to practices within randomly assigned regions in three distinct steps (26-30 practices per step), with each consecutive step (or cohort of practices) beginning the intervention approximately one year apart. The multifaceted IDOCC intervention, which is based on the chronic care model, is being offered over a 24-month period. The primary outcome is a composite score measured at the level of the patient to examine each practice's adherence to evidence-based guidelines for cardiovascular disease care. IDOCC has received ethical approval from the Ottawa Hospital Research Ethics Board [8].

### Study Setting

This study was conducted in the Champlain Region of Eastern Ontario, which encompasses the city of Ottawa and surrounding rural communities, totalling over 1.2 million people [10,11]. This region has chronic disease burdens and patient health outcomes that are comparable to the rest of Ontario and Canada [10,11].

### Practice Recruitment

All models of primary care practices in the selected geographic areas of the Champlain region, excluding walk-in clinics, were eligible to participate in this project. At the time of the initiation of this study (January 2008), there were 823 family physicians offering comprehensive care in 533 practices in this region, including: fee for service practices, family health groups, community health centres, family health networks, family health organizations and family health teams. The characteristics of each model are summarized in Table 1[7].

**Table 1:** Comparing the features of primary care models in Ontario, Canada.

Characteristic	Salary	FFS		Blended Capitation		
	
	Community Health Centre (CHC)	Fee For Service (FFS)	Family Health Group (FHG)	Family Health Network (FHN)	Family Health Organization (FHO)	Family Health Team (FHT)
Year introduced	1970s	......	2004	2001	2006	2005

Group size	Group practice, size unspecified	1 Physician	Minimum of 3	Minimum of 3	Minimum 3	Group practice, size unspecified

Physician remuneration	Salary	FFS	FFS + incentives	Blended Capitation^b^	Blended Capitation^b^	Blended Capitation^b^

Patient enrollment	Required,	Not required	Required	Required	Required	Required

Access	Extended office hours	No specified requirements	Extended office hours, THAS	Extended office hours, THAS	Extended office hours, THAS	Extended office hours, THAS

Multidisciplinarity^a^	Extensive	None	None	Some	Some	Extensive

Table adapted from Russell et al. [7]. THAS = Telephone Health Advisory Service, a patient telephone advisory system for which physicians are required to provide on-call services 24 hours a day, 7 days a week

^a ^Multidisciplinarity refers to the presence of allied health professionals (e.g., physiotherapist, social worker, dietician, pharmacist), excluding nursing staff, but including nurse practitioners

^b ^Blended Capitation - a base payment (adjusted for age and sex) for all enrolled patients is provided to physicians for the provision of comprehensive care along with incentives, premiums and special payments for the provision of specific primary health care services.

Practice recruitment was carried out using a modified Dillman approach involving reminders and repeat mailings [12]. Practices were enlisted into the program if at least one physician agreed to participate in IDOCC. Each practice was asked to sign a consent form allowing the project team to collect information from their patient medical charts. Recruitment efforts for each step continued until consent was obtained from 27 practices per step. This sample size was based on the requirements for the IDOCC project.

We categorized each practice into one of three groups:

1. Fee for service (FFS): Practices were classified as FFS if physicians receive the majority of payment through fee for service billing. This group includes traditional FFS practices and the reformed FFS model, the family health group (FHG). FHGs are paid primarily through fee for service payments, however, they also receive incentive and premium payments for patient enrolment, health promotion activities, and for the management of certain conditions (e.g., diabetes care incentives). Physicians in this group do not receive any governmental support to hire allied health providers or to set up an electronic medical record (EMR) system.

2. Blended Capitation: Practices were classified in this group if physicians are principally paid through capitation. Physicians in this model receive a base payment (adjusted for age and sex) for each enrolled patient for the provision of comprehensive care and they also receive a small portion of their salary from FFS billing. Physicians are also given incentives, premiums and special payments for the delivery of specific primary health care services (e.g., diabetes care, smoking cessation counselling, etc). This group includes family health networks, family health organizations and family health teams, of which, all family health teams receive governmental support to hire allied health professionals and to set up an EMR system, while some family health organizations employ allied health professionals through governmental grants.

3. Community Health Centres (CHCs): CHCs are community-governed organizations that are made up of large interprofessional health care teams. Physicians practicing in CHCs are paid a fixed annual salary irrespective of the number or nature of services they provide. CHCs typically serve in underserviced, low-income populations and regularly offer a series of health promotion programs for their communities.

In cases where a practice shifted from one group to another during the baseline data collection timeframe, we assigned that practice to the group in which they spent the majority of the year in.

### Data Collection

We used patient medical chart audits to examine each practice's adherence to recommended evidence-based best practice manoeuvres for cardiovascular care. We assessed whether recommended manoeuvres were performed, recommended, or discussed during the one year preceding the abstraction date. Since the delivery of the IDOCC program was staggered in the stepped wedge implementation approach, the baseline data collection timeframes varied among steps: Step I: 2007-2008, Step II: 2008-2009, and Step III: 2009-2010.

Six trained chart abstractors collected data on specific process of care indicators based on recommendations from The Champlain Primary Care Cardiovascular Disease Prevention and Management Guideline. This guideline was developed by an expert panel of family physicians and specialists (e.g., cardiologists, endocrinologists, etc) who critically reviewed and harmonized current national and international guidelines for cardiovascular disease and its associated risk factors [13]. The guideline can be viewed at http://www.idocc.ca.

To ensure the consistency and quality of the abstracted data across chart abstractors, a four-part quality-monitoring process was established, which includes (1) standardized protocol implementation, (2) extensive data abstraction training, (3) continuous re-abstraction and validation to monitor the interrater reliability between Abstractors, and (4) constant feedback and retraining. Our overall baseline interrater reliability kappa value was 0.91, and the overall percent agreement was 94.3% [14].

### Patient Eligibility Criteria

Charts were eligible for this study, if the following patient inclusion criteria were met: over the age of 40, resident of Ontario, patient of a physician who consented to take part in IDOCC, having been seen at least once within the abstraction timeframe and had an overall record in the practice going back at least two years, having at least one of the following: established cardiovascular disease (i.e., coronary artery disease, stroke and/or transient ischemic attack, or peripheral vascular disease); diabetes mellitus; chronic kidney disease; or are at high risk for cardiovascular disease based on the presence of at least **three **of the following four established cardiovascular risk factors: age (males ≥ 45, females ≥ 55), smoker status, hypertension, and dyslipidemia.

### Outcome Measures

We examined adherence to ten predefined evidence-based guidelines in six areas of care: dyslipidemia, diabetes, chronic kidney disease, hypertension, weight management, and smoking cessation care. For example, for smoking cessation care, we examined whether a patient was recommended a smoking cessation drug, received counselling from a health care provider, or was referred to a help program. Full details on the ten processes of care indicators are listed in Table 2.

**Table 2:** Process of care indicators

Area of care	Process of care Indicator*	Eligible patients**	Description
Dyslipidemia	Lipid profile	All patients	Method of measuring blood cholesterol levels
	Lipid lowering drug	Dyslipidemia	Prescribed to reduce levels of LDL cholesterol ('bad' cholesterol)

Diabetes	2 Hemoglobin A1c (HbA1c) tests	Diabetes	Measures average blood sugar level over past 3-4 months

Chronic kidney disease	Estimated glomerular filtration rate (eGFR)	Chronic kidney disease	Used to assess kidney function

Hypertension	2 blood pressure measures	Hypertension	Hypertension is a risk factor for heart disease & stroke
	Anti-hypertensive drug		Taken to reduce blood pressure i.e. ACE Inhibitors, Beta Blockers

Weight	Waist circumference measure	All patients	Waistline is a strong predictor of cardiovascular disease-related risk factors, all cause mortality, and Type II diabetes

	Smoking cessation advice		
Smoking cessation	Smoking cessation program referral	Smokers	Physician advice and cessation programs strongly impact patient smoking status
	Smoking cessation drug therapy		i.e. Nicotine replacement therapies

*Was the following process of care indicator discussed/recommended/performed during the past 12 months for this patient ** The eligibility for recommended processes of care varies for each patient depending on current diagnoses. For example, monitoring HbA1c twice within a year is only recommended for patients with diabetes.

### Data Analysis

Descriptive statistics were generated for all study variables and practice/physician characteristics. Chi-square tests were used to compare practice level characteristics amongst the three models. In cases where expected counts were less than 5, Fisher's exact test was used. Patient-level characteristics were compared amongst the three models while adjusting for clustering within a practice using generalized estimating equations for dichotomous variables and mixed models for continuous variables.

Dichotomous outcome variables examining whether one of the ten pre-defined process of care indicators was performed using a generalized estimating equation, accounting for clustering of patients within practices using an exchangeable correlation structure. The primary independent variable of interest in this analysis was practice payment model, analyzed as a three level categorical variable. We adjusted for the following patient and practice characteristics as literature suggests that they are important in predicting quality of care: patient age, patient sex, rurality, number of cardiovascular-related comorbidities, and year of data collection [7,15-18]. The statistical significance of the differences among the primary care models were assessed using pairwise comparisons at the Bonferroni-corrected significance level of 1.7% (adjusted for multiple comparisons). All analyses were conducted using SAS, Version 9.2, SAS Institute Inc. [19].

## Results

### Characteristics of Study Population

We approached all 533 primary care practices in the Champlain region to participate in the IDOCC program. Ninety nine were ineligible to participate because the practice was no longer in operation, the clinic was an exclusive walk-in practice, or the physician running the practice was planning to retire within the two year intervention timeframe. Of the 434 eligible practices, 91 practices agreed to participate, of which nine dropped out prior to baseline data collection.

Of the 82 practices, 52% (n = 43) were FFS, 33% (n = 27) were blended capitation, and 15% (n = 12) were CHCs. Baseline characteristics varied across models (Table 3), with CHCs being exclusively interprofessional teams, while both blended capitation and CHCs had a higher percentage of practices that used electronic medical records when compared to FFS practices. Patient profiles were similar across the models (Table 3).

**Table 3:** Breakdown of practice/patient level characteristics by payment model

	Primary care payment model			p-value
		
Characteristics	FFS	Blended Capitation	CHC	
**Practice Level Characteristics**				
Number of practices (n = 82)	43	27	12	-
Step I	20 (80%)	1 (4%)	4 (16%)	-
Step II	12 (40%)	12 (40%)	6 (20%)	
Step III	11 (41%)	14 (52%)	2 (7%)	
Multidisciplinary practices*	2 (5.0%)	10 (37%)	12 (100%)	< 0.0001
Practices using EMR	7 (16%)	21 (78%)	11 (92%)	< 0.0001
Urban practices^+^	36 (84%)	23 (85%)	8 (67%)	0.35
Physician graduation year (median, IQR)	1983 (11)	1984 (9)	1991 (4.5)	-
**Patient Level Characteristics**				
Number of patients (n = 4808)	2565	1555	688	-
Patient age (mean, SD)	66 (11.5)	66 (11.4)	64 (11.9)	0.0002
Female patients (n, %)	1356 (53%)	757 (49%)	354 (51%)	0.47
Number of cardiovascular disease-related comorbidities per patient (Mean, SD)	2.7 (1.1)	2.8 (1.0)	2.8 (1.1)	0.20
Diabetes (n, %)	1191 (46%)	734 (47%)	332 (48%)	0.82
Chronic kidney disease (n,%)	457 (18%)	294 (19%)	117 (17%)	0.61
Dyslipidemia (n, %)	2135 (83%)	1314 (85%)	591 (86%)	0.40
Hypertension (n, %)	1955 (76%)	1194 (77%)	554 (81%)	0.35
Smokers (n, %)	514 (20%)	324 (21%)	202 (29%)	0.07

* Multidisciplinary refers to presence of allied health professionals (ie. social worker, dietician, pharmacist), excluding nurse staff, but including nurse-practitioners.

+ Based on Statistics Canada definition of urban areas (ie. 'An urban area has a minimum population concentration of 1,000 persons and a population density of at least 400 persons per square kilometre')

### Comparison of Care Delivery across Primary Care Payment Models

Table 4 summarizes the percentage of eligible patients from each model who received the process of care manoeuvres, while unadjusted and adjusted comparisons between models are presented in Tables 5 and 6 respectively. We found differences in care amongst the three models in the areas of diabetes management, smoking cessation care, and weight management, while there was little variability in care across models for chronic kidney disease, dyslipidemia, and hypertension management.

**Table 4:** Unadjusted adherence to process of care indicators

Area of care	Process of care indicator	% of patients that received specified process of care Indicator		
		
		FFS	Blended Capitation	CHC
Dyslipidemia	Lipid profile	78% (1995/2565)	81% (1255/1555)	78% (539/688)
	
	Lipid lowering drug	92% (1961/2136)	92% (1206/1314)	90% (529/591)

Diabetes	2 HbA1c tests	45% (538/1191)	62% (453/734)	69% (229/332)

Chronic Kidney Disease	eGFR	91% (415/457)	93% (273/294)	91% (107/117)

Hypertension	2 blood pressure measures	78% (1531/1955)	79% (945/1194)	81% (448/554)
	
	Anti-hypertensive drug	95% (1850/1955)	94% (1119/1194)	94% (518/554)

Weight	Waist circumference measure	5% (117/2565)	19% (294/1555)	8% (58/688)

	Smoking cessation advice	42% (214/514)	67% (216/324)	56% (113/202)
	
Smoking Cessation	Smoking cessation program referral	5% (27/514)	11% (37/324)	8% (16/202)
	
	Smoking cessation drug therapy	19% (98/514)	33% (107/324)	16% (33/202)

**Table 5:** Unadjusted comparison of process of care indicators between primary care payment models

Area of care	Process of care indicator	p-value	Pairwise comparison of models: OR [95% CI]		
			
			Blended Capitation vs. FFS	CHC vs. FFS	Blended Capitation vs. CHC
Dyslipidemia	Lipid profile	0.30	1.2 [0.9-1.6]	1.0 [0.7-1.4]	1.2 [0.8-1.7]
	Lipid lowering drug	0.60	1.0 [0.7-1.5]	0.8 [0.5-1.2]	1.3 [0.8-2.3]

Diabetes	2 HbA1c tests	0.0008*	1.9 [1.3-2.8]^†^	2.5 [1.4-4.4]^†^	0.8 [0.4-1.4]

Chronic Kidney Disease	eGFR	0.66	1.4 [0.7-2.7]	1.1 [0.4-3.1]	1.3 [0.4-4.1]

Hypertension	2 blood pressure measures	0.80	1.1 [0.7-1.7]	1.2 [0.8-1.8]	0.9 [0.6-1.4]
	Anti-hypertensive drug	0.70	0.9 [0.6-1.3]	0.8 [0.5-1.4]	1.0 [0.6-1.8]

Weight	Waist circumference	0.003*	4.8 [2.4-9.7]^†^	1.9 [0.8-4.3]	2.6 [1.1-5.8]

	Smoking advice	0.001*	2.6 [1.6-4.3]^†^	1.6 [0.9-3.0]	1.6 [0.9-3.1]
Smoking Cessation	Smoking program referral	0.20	2.5 [1.0-6.2]	1.4 [0.6-3.3]	1.8 [0.7-4.3]
	Smoking drug therapy	0.005*	2.1 [1.3-3.3]^†^	0.8 [0.4-1.6]	2.7 [1.5-4.9]^†^

* p < 0.05: Indicates that payment model has a significant effect on the delivery of the specified indicator

^† ^p < 0.017: p-value limit for between group comparisons was corrected using a Bonferroni correction factor

OR = Odds Ratio, CI = 95% confidence interval on odds ratio

**Table 6:** Adjusted comparison of process of care indicators between primary care payment models

Area of care	Process of care indicator	p-value	Pairwise comparison of models: AOR [95% CI]		
			
			Blended Capitation vs. FFS	CHC vs. FFS	Blended Capitation vs. CHC
Dyslipidemia	Lipid Profile	0.60	1.1 [0.9-1.4]	0.9 [0.7-1.3]	1.2 [0.8-1.7]

	Lipid lowering drug	0.24	0.8 [0.5-1.3]	0.7 [0.4-1.0]	1.2 [0.7-2.1]

Diabetes	2 HbA1c tests	0.01*	1.5 [1.1-2.3]	2.4^†^ [1.4-4.2]	0.6 [0.3-1.2]

Chronic Kidney Disease	eGFR	0.90	1.2 [0.6-2.3]	1.1 [0.4-3.2]	1.1 [0.3-3.9]

Hypertension	2 Blood Pressures	0.73	1.0 [0.6-1.5]	1.2 [0.7-1.8]	0.9 [0.5-1.3]
	Antihypertensive drug	0.25	0.7 [0.4-1.0]	0.9 [0.5-1.3]	0.8 [0.5-1.4]

Waist Circumference	Waist Circumference (WC)	0.007*	3.7 ^†^ [1.8-7.8]	2.0 [1.0-4.2]	1.8 [0.8-4.0]

	Smoking advice	0.13	1.6 [1.0-2.6]	1.5 [0.8-2.9]	1.0 [0.5-2.1]
Smoking Cessation	Smoking Program	0.25	2.7 [0.8-9.2]	1.1 [0.4-2.6]	2.6 [0.9-7.3]
	Smoking drug therapy	0.04*	1.5 [0.9-2.5]	0.6 [0.3-1.1]	2.4 ^†^ [1.3-4.6]

Adjusted for age, sex, rurality, number of cardiovascular-related comorbidities, and year of data collection

* p < 0.05: Indicates that payment model has a significant effect on the delivery of the specified indicator

^† ^p < 0.017: p-value limit for between group comparisons was corrected using a Bonferroni correction factor

AOR = Adjusted Odds Ratio, CI = 95% confidence interval on odds ratio

Diabetes Management: Adherence to HbA1c guidelines (i.e., two HbA1c tests within a year) was highest in CHCs, as 69% of patients received two HbA1c tests during the examination year, compared to 62% and 45% in blended capitation and FFS practices, respectively (Table 4). Adjusted comparisons among payment models demonstrated that CHCs were significantly more likely to follow HbA1c monitoring guidelines than FFS practices (Adjusted Odds Ratio (AOR) = 2.4, 95% CI 1.4 to 4.2) (Table 6). Unadjusted results demonstrated that blended capitation practices were also significantly more likely to follow HbA1c guidelines than FFS practices, however, pairwise comparisons in the adjusted model just came short of reaching statistical significance at the stringent Bonferroni-corrected level α = 0.017 (AOR = 1.5, 95% CI 1.1-2.3, p = 0.026). Nevertheless, the adjusted analysis supports the unadjusted analysis suggesting that blended-capitation practices tend to have higher odds of following HbA1c guidelines than FFS practices.

Smoking Cessation Care: Adjusted comparisons demonstrated that smokers being treated in blended capitation practices had a significantly higher chance of being recommend smoking cessation drug therapies than patients in CHCs (AOR = 2.4, 95% CI 1.3 to 4.6) (Table 6). Furthermore, while the unadjusted comparisons suggested that blended capitation practices might be more likely to provide smoking cessation counselling (Table 5), these differences were not significant after adjustment (Table 6). Adjusted analyses demonstrated that year of data collection had a significant impact on smoking advice, as practices that had their charts abstracted in 2007-2008 were less likely to give their smoking patients advice than those who had their data collected during 2008-2009 (AOR = 2.4, 95% CI 1.5 to 3.9, p = 0.0002) and 2009-2010 (AOR = 3.8, 95% CI 2.2 to 6.7). Improved smoking cessation counselling was seen when examining each model individually, thus indicating that these observed trends were in fact due to improvements over time and not because of the imbalances of models in each step (i.e., 20/26 practices that got their data abstracted in 2007-2008 were FFS).

Weight Management: FFS practices had the poorest adherence to waistline measuring guidelines, as only 5% of all patients received a waistline measurement, compared to 8% in CHCs and 19% in blended capitation practices (Table 4). Within the blended capitation group, FHTs had the highest adherence rate, as 28% of patients within these practices received a waistline measurement. Adjusted analyses demonstrated that waist circumference monitoring was significantly higher in practices operating with a blended capitation payment model when compared to FFS practices (AOR = 3.7, 95% CI 1.8 to 7.8) (Table 6).

Adherence to guidelines in the areas of chronic kidney disease, dyslipidemia, and hypertension management were all above 75% in each model, with no significant differences being observed across models for any of these indicators (Table 4).

## Discussion

We have found important differences in the quality of cardiovascular care delivery among existing primary care models. Our findings demonstrate that blended capitation practices such as family health teams provided superior care in the areas of smoking cessation care and waist circumference management, while HbA1c monitoring was highest in CHCs. FFS practices had the greatest gaps in care within this study, most noticeably in the areas of diabetes care, and waist circumference management.

Patients being treated in CHCs had 2.4 times the odds of receiving recommended HbA1c screening than those in FFS practices. Regular monitoring of HbA1c is essential in assessing glycemic control in patients as it has been shown to be a strong predictor of various diabetes-related and cardiovascular complications [20].

Our results are similar to findings from another Ontario-based study, which demonstrated that CHCs were superior in delivering high quality diabetes care when compared to FFS practices, as they also had a significantly higher proportion of patients who received regular foot and eye examinations and appropriate HbA1c screening [7]. Several studies conducted in the United States have demonstrated that practices that primarily pay their physicians through direct salary provide strong diabetes care [21-23]. The CHC's payment structure, organizational structure and care delivery approach which includes team care, focused clinics and the use of an EMR is likely a strong contributor to the observed differences in quality of diabetes care delivery.

In general, smoking cessation care was strongest in practices operating under a blended capitation payment model. Blended capitation practices were significantly more likely to prescribe smoking cessation drugs to their patients than CHCs and also had the highest rates of smoker counselling and referral to smoking cessation programs. These relatively high adherence rates were due in part to the high performance of the FHTs within the blended capitation group. For example, 25% of the smokers in FHTs were referred to a smoking cessation program, while the FHNs and FHOs (i.e., the other two models in the blended capitation group) had a combined adherence rate of only 7%. This substantial variability along with the relatively small sample size of smokers in this study likely contributed to the fact that the differences in adherence to this process manoeuvre were not found to be significant in the analysis.

The quality of care delivery for smoking cessation in the blended capitation group may be attributable to the fact that the FHTs are comprised of multidisciplinary teams that often hold smoking cessation clinics or programs. In the case of CHCs, many centres also run smoking cessation clinics, and previous studies have demonstrated that they provide superior health promotion [24]. Despite the above, CHCs had the lowest adherence rates for recommending smoking cessation drugs, a finding that may be due to the fact that many patients in CHCs are of low socioeconomic standing, and thus, they may be unable to afford nicotine replacement therapies and other smoking cessation drugs [24,25].

We also found that smoking counselling improved over time. This finding may be a result of increased awareness about the importance of physician counselling in helping patients quit, or potentially due to the rise in smoking campaigns in Eastern Ontario during the study timeframe [26].

Studies have shown that combining nicotine replacement therapy with counselling doubles ones chances of successfully quitting [27]. It is anticipated that with stronger smoking cessation care in the blended capitation group, these practices will potentially have a patient population that is more likely to quit smoking than their counterparts who visit other practice models; thereby the overall health outcomes of the blended capitation patients will improve.

The adoption of waist measurement was low in all models, ranging from 5-19%. We did find that patients in blended capitation practices were significantly more likely to receive a waist circumference measurement when compared to patients in FFS practices. Waistline measurement is important as it has been shown to be a strong predictor for cardiovascular disease related risk factors (i.e., hypertension, dyslipidemia), all cause mortality, and type II diabetes [28,29]. Waist circumference measures provide an independent estimate of patient health risks independent of body mass index (BMI) [28]. With an increased monitoring of patient waistlines, blended capitation practices are more likely to identify high-risk patients, which may lead to improved weight management and patient clinical outcomes.

This low adherence rate to the guidelines is a good example of how long it takes to get evidence-based care into practice, as the importance of waistline monitoring has been recommended in obesity guidelines as early as 1998 [30]. Previous reports have shown that it can take up to seventeen years for a relevant research discovery to be incorporated into primary care practices [31]. Through the IDOCC program, we are trying to accelerate knowledge translation in primary care using an outreach facilitation approach, in which an external healthcare professional with training in facilitating practice change, visits community practices to assist physicians and staff in improving their delivery of evidence-based care. Early evidence suggests that facilitation can be an effective means of enhancing primary care providers adoption of evidence based guidelines [32].

Although the above findings demonstrate that there are significant differences in care amongst the three models, it is difficult to definitively say which organizational factors resulted in the differences. Practices within the same model share common organizational factors such as charting system, team structure and remuneration model, and thus, it is not possible to pinpoint which of these factors led to the differences in care observed in this study. Previous studies have shown that remuneration model, team structure, information technology infrastructure, and physician panel size all have an impact on quality of care [7,33-35]. Thus, the findings observed in this study are most likely due to a combination of various factors. Adding to the complexity of assessing quality of care is the fact that all practices have individualized characteristics which influence care delivery. For example, when comparing adherence to HbA1c guidelines in two FHTs within our study, one practice had an adherence rate of 85% while the other had a rate of 36%. Despite sharing the same payment structure, EMR charting system, and similar team structures, HbA1c screening in both practices is noticeably different. These variations are likely due to individualized practice-level characteristics such as the overall culture within the practice [36] (i.e., quality centered or business oriented, autonomous), practice organization, degree of interprofessional collaboration, and variability's in office systems approach.

### Study Limitations

This study has several limitations. Firstly, this was a secondary analysis of data collected from a study designed to improve overall cardiovascular disease care with primary care practices using an outreach facilitation model, and thus, was not specifically designed to compare quality of care across models. An ideal study design to compare models would include stratification by model to ensure equal representation. In this study, the imbalance by model meant that we had greater power to detect changes between certain groups compared to others. Secondly, the low recruitment rate and self selection into the IDOCC program may impact the generalizability of our findings. Participants in this study are highly motivated and their quality of care is likely higher than the average primary care provider. As such, levels of adherence to guidelines found in this study were likely higher than averages across the province limiting the effect size as well as the generalizability of our findings. However, the same bias applied to providers in all three models, and thus, this should not impact our comparisons and conclusions. Thirdly, the small sample size within certain patient subgroups limited our ability to detect statistical significance for specific comparisons that appeared to be clinically meaningful differences (i.e., smoking program referral).

Lastly, since we relied on chart abstraction to ascertain care levels, activities that were performed but not charted would not have been captured. However, chart abstraction remains the gold standard for capturing process of care data, as the alternative, direct observation, is prohibitively expensive and not feasible for large trails [37]. Since the same data collection approach was used across all models, this limitation should not impact our conclusions.

## Conclusions

The aim of this study was to compare the performance of primary care models in delivering cardiovascular care. This study adds to the evidence suggesting that primary care model influences quality of care. Our findings demonstrate that diabetes care was superior in CHCs, while smoking cessation care and weight management was superior in the blended-capitation models. These findings support current Ontario reforms to move away from the traditional FFS practice. Chronic disease care is a complex, multi-faceted, and a time consuming process, and as such, patients with these conditions need greater attention and require care from a diverse group of health care professionals. Despite evidence that blended capitation and CHCs provide a higher level of care, future research needs to focus on examining the population health and economic impact of incorporating these primary care models into healthcare systems.

## Competing interests

The authors declare that they have no competing interests.

## Authors' contributions

CL, JS, and SD all contributed to the conception of the idea to carry out this sub-study to compare the quality of care amongst primary care models. CL and WH originally conceived and designed the IDOCC study. JS and MT formulated and carried out the data analysis plan for this sub-study. All authors read and approved the final manuscript.

## Pre-publication history

The pre-publication history for this paper can be accessed here:

http://www.biomedcentral.com/1471-2296/12/114/prepub
